# The Treatment of Corneal Ulcers

**Published:** 1892-10-15

**Authors:** 


### The Hospital Clinic.

[*The Editor will be glad to receive offers of co-operation and contributions from members of the profession. All letters should be addressed to* The Editor, The Lodge, Porchester Square, London, W.]

### MOORFIELDS OPHTHALMIC HOSPITAL.

### The Treatment of Corneal Ulcers.

Ulcers of the cornea are of such common occurrence that their treatment must be of considerable interest. The chief drug used in the treatment of corneal ulcers used to be atropine, now eserine very largely replaces it. It is used generally as a very weak solution, half or a quarter of a grain to the ounce, but some prefer it as strong as two to four grains to the ounce, and say this strength does good when the weaker solution fails; but eserine of this strength generally causes much dragging pain in the eye when used. The eserine is sometimes combined with a two per cent. solution of cocaine. This, of course, is specially useful when photophobia is a marked symptom. Eserine is supposed to act by increasing the blood supply, and also, perhaps, by reducing tension. It is, therefore, not indicated in ulcers to which a leash of vessels are running, as there is no need to increase their vascularity. But cocaine certainly constricts the vessels, and hence would neutralise the effect of the eserine when they were combined, unless the effect of the eserine lasted much longer than cocaine. We hardly think the increase of vascularity quite a sufficient explanation of the utility of eserine in corneal ulcers.

When the ulcer is indolent the eserine drops may be used with an ointment of the yellow oxide of mercury, of the strength of eight grains to the ounce. Mr. Tweedy uses atropine and not eserine for these ulcers. Mr. Gunn never uses yellow ointment. If atropine is used an ointment of four grains of atropine and eight grains of yellow oxide of mercury to the ounce does well. Atropine, especially when combined with hot fomentations, is apt to set up atropine irritation in the form of a dry eczema about the lids, but the atropine ointment does not seem to act in this way, and is generally preferred.

Besides the eserine frequent bathing with hot boracic acid solution is employed. Each patient when treated in the hospital has his bowl of boracic or perchloride solution stewing on the stove, and every now and again bathes the eye with a mass of cotton wool, which is kept in the hot fluid, and acts as a sponge. The eyes are not tied up with hot fomentations at Moorfields. To exclude the light, a shade or dark glasses are worn when necessary. The perchloride solution used is of the strength of 1 in 5,000; the boracic about 2 per cent.

Sometimes an injury starts ulceration, which goes on spreading, and develops irregular infiltrated edges with effused lymph. In such cases eserine and hot bathing may be first tried, and if this fails to start the healing process the treatment employed is the galvano-cautery at a dull red heat all round the margin of the ulcer after the application to it of some solid cocaine. The solid cocaine seems to be frequently used. If it cannot be very readily determined how far the ulceration extends or whether it is beginning to heal and is diminishing in size, fluorescine proves of great value. When dropped into the eye, it is found to stain green any surface denuded of its epithelium. It thus marks out exactly the area of the ulcer, so that thorough cauterisation of its margin can be made, and the rate of its healing, and the need of further cauterisation can be judged from time to time. Mr. Morton has found fluorescine very useful in this way in the treatment of corneal ulcers, and also in cases where a minute chip of glass or other foreign body not easily seen is embedded in the cornea, as a means of differentiating between the foreign body and the cornea, immediately around which it stains green, and thus shows up the foreign body. In this way it is more easy to be sure when the last particle of a foreign body embedded in the cornea has been removed.

Ulcers of the cornea complicated with hypopyon, are generally cauterised in the way described, and then eserine and hot bathing are used. If the ulcer spreads it is cauterised again. If it spreads and the hypopyon increases, in elderly persons, the anterior chamber is tapped by a broad needle, and the pus evacuated. Sometimes the exudation is more lymph than pus, and then has to be drawn out with iridectomy forceps. Sometimes these hypopyon ulcers when they increase, are treated by making a section of the cornea, through the ulcer. A narrow knife is passed under the ulcer and made to cut its way out right through its base. This method was recommended by Saemish, and is known as Saemish's section. It is not often employed at Moorfields, as adhesion of the iris to the cornea at the seat of section (anterior synechia) is so apt to follow, a condition serious to vision, but also rendering the affected eye liable to set up sympathetic trouble in the other.

There are some cases of so-called strumous ophthalmia in children, cases where phlyctenular ulcers occur on the cornea, and there is much swelling and redness of the conjunctiva, with swollen lids and generally intense photophobia. These cases are best treated also with eserine and bathing with hot boracic acid solution, and some surgeons find ten grains to the ounce nitrate of silver solution brushed over the inflamed palpebral conjunctiva every day of service. This is generally washed off again at once with salt solution, but all surgeons do not adopt this practice, and it is said that no harm results from leaving the nitrate of silver solution on; they do not even wash it off with water.

It is hardly ever found necessary to divide the outer canthus for photophobia. The use of cocaine and eserine relieves this without. The plan of ducking the child's face into a basin of water is said to have more than a momentary effect on the photophobia, and the habit thus broken at the time is found often to be permanently diminished.

Mr. Morton describes a crescentic ulcer which tends to spread round the cornea and cause the centre to slough. In these cases he has found eserine very useful treatment. He never uses it stronger than ¼ grain to the ounce for any form of ulcer.

Little need be said about the internal administration of drugs. Cod liver oil and the syrup of iodide of iron are given to "strumous" children, and tonics to debilitated adults. Country air is very useful in these "strumous" patients; but when they are sent away too soon they are apt to get worse rather than better, from the want of proper directions and supervision in carrying out local treatment.

